# Cross-cultural adaptation and validation of the Arabic version of the Malocclusion Impact Scale for Early Childhood (MIS-EC/Ar)

**DOI:** 10.1038/s41405-026-00411-6

**Published:** 2026-04-13

**Authors:** Ahmed Kamal El-Motayam, Rim Fathalla, Marwah Salah Abdel-Latif, Iman Ali El-Baraky, Lamia Khairy Gadallah

**Affiliations:** 1 Department of Pediatric Dentistry and Dental Public Health, Faculty of Dentistry, Cairo University, Cairo, Egypt; 2 Department of Orthodontics, Faculty of Dentistry, Suez Canal University, Ismailia, Egypt; 3 Department of Orthodontics, Faculty of Dentistry, Ain Shams University, Cairo, Egypt; 4 Department of Clinical Pharmacy, Faculty of Pharmacy, Cairo University, Cairo, Egypt; 5 Department of Orthodontics and Pediatric Dentistry, National Research Center, Cairo, Egypt

**Keywords:** Tooth brushing, Occlusion

## Abstract

**Background:**

Malocclusion is a highly prevalent oral disease in children. It could detrimentally affect their oral health-related quality of life (OHR-QoL). The Malocclusion Impact Scale for Early Childhood (MIS-EC) is a newly developed scale for the assessment of OHR-QoL in this population aged 3–5 years-old. The aim of this study is to translate and validate an Arabic version of the MIS-EC scale (MIS-EC/Ar).

**Materials and methods:**

Translation and cross-cultural adaptation of the MIS-EC/Ar scale were performed conforming to relevant WHO guidelines. A sample of mothers/caregivers of children aged between 3 and 6 years was recruited (*n* = 236). Clinical examination of children and assessment of malocclusion were performed before taking MIS-EC/Ar. The internal consistency was assessed using Cronbach’s alpha. Test-retest reliability performed after two weeks (*n* = 30) employed intraclass correlation coefficient (ICC). Construct validity, including convergent, discriminant, and structural validity, was evaluated in addition to criterion validity.

**Results:**

MIS-EC/Ar showed good internal consistency (standardized Cronbach’s alpha = 0.797) and excellent test-retest reliability (ICC = 0.929, 95% CI 0.884–0.962). Convergent validity, Spearman correlation coefficients for the MIS-EC/Ar total score with the overall oral health question, was limited (*r* = 0.159, *P* < 0.05). Discriminant validity was significant (*p* < 0.05). Criterion validity was fair (*r* = 0.298, *p* = 0.01). Exploratory factor analysis suggested a reduced scale of six items.

**Conclusions:**

The MIS-EC/Ar is a reliable instrument with good discriminant validity for assessing malocclusion impact on preschool children’s OHR-QoL. Limited convergent validity and altered factor structure suggest cultural adaptation effects.

## Introduction

Malocclusion is one of the most common oral health diseases following both dental caries and periodontal diseases [1]. Malocclusion is a multifactorial condition influenced by both genetic and environmental factors, characterized by abnormal dental-maxillofacial growth and development [2]. It is defined as “an abnormal occlusion in which teeth are not in a normal position in relation to adjacent teeth in the same jaw and/or the opposing teeth when the jaws are closed” [3]. The worldwide prevalence rates of malocclusion are relatively high in both children and adolescents, reaching 56% [4]. The occlusal features of malocclusion in children include sagittal anomalies as increased overjet or anterior crossbite, vertical anomalies as deep overbite or anterior openbite, and transversal anomalies as posterior crossbite in addition to spacing problems [5–9]. Health care questionnaires can measure different items, including symptoms, functioning as well as the effect of the disease on general health or quality of life [10]. As a consequence to the developed definition of health from the World Health Organization (WHO) in the second half of the 20th century, the oral health-related quality of life (OHR-QoL) has emerged as a concept to measure the effect of oral conditions not only on the physical level, but also on the psychological and social levels [11].

There are several tools that are used to assess the OHR-QoL in both adolescents and children, addressing the impact of caries, trauma, and malocclusion [12–14]. However, the tools specified for preschool children were very few, and none of them were specified to assess the impact of malocclusion in this young age [15, 16]. The scales available are mainly concerned with the effect of early childhood caries [17–19].

Recently, the Malocclusion Impact Scale for Early Childhood (MIS-EC) was developed and validated as a tool for assessing the OHR-QoL of children aged from 3 to 5 years with their parents/caregivers concerning malocclusion [20]. The data on prevalence rates of malocclusion in children of Arabic countries is scarce [21–23]. However, a recent report showed high rates of malocclusion in preschool children in Saudi Arabia [24].

An Arabic version of the scale can be beneficial for assessing the impact of malocclusion on OHR-QoL in Arabic-speaking preschool children across the Arab region.

The aim of this study is to translate and validate the MIS-EC scale in the Arabic language and to evaluate the impact of malocclusion on OHR-QoL in Arabic-speaking preschool children.

## Materials and methods

### Study design

**Fig. 1:**
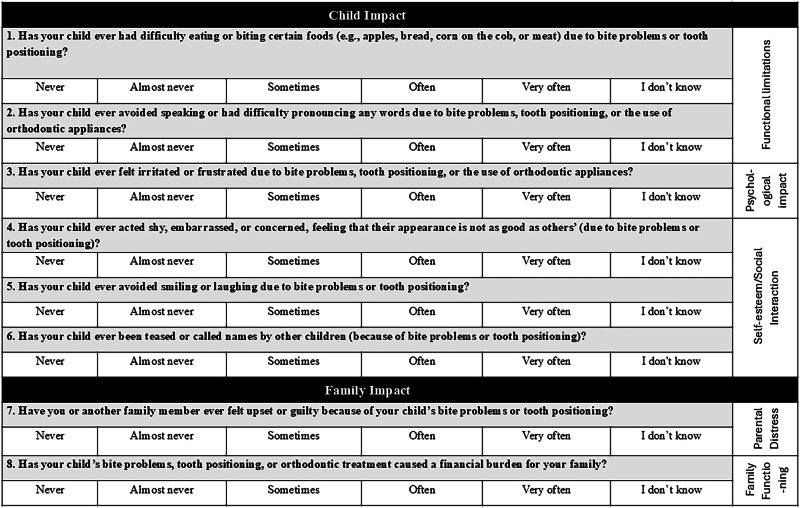
The English version of the Malocclusion Impact scale for Early childhood. The scale consists of two sections: the child impact section included functional, psychological, social and self-esteem domains and the family impact section included parental distress and the family functioning domains.

MIS/EC was translated into Arabic language then undergone the steps of cross-cultural adaptation and validation to provide a reliable tool to assess parental perceptions of the effect of malocclusion on OHR-QoL in preschoolers. The scale is shown in Fig. 1. The study followed the Declaration of Helsinki guidelines. This study was approved by the Research Ethics Committee of the Faculty of Dentistry, Cairo University, Cairo, Egypt, with reference number 46/9/24. Participants provided informed consent after being fully explained the study details and purpose.

### Original MIS-EC questionnaire

The original MIS-EC was developed and validated by Homem et al. in both English and Brazilian Portuguese languages [20]. The English version was obtained through contacting the authors of the original questionnaire. It includes eight questions divided into two sections/domains; the first section is composed of six questions about the child impact. The second section is composed of two questions about the family impact. The child impact section examined functional, psychological, social, and self-esteem impact of malocclusion. The family impact section examined parental distress and the family functioning concerning the financial impact.

MIS-EC scoring adopted a Likert scale with scores (zero–four) denoting the corresponding frequency of (never–very often) in addition to a response of “I don’t know”. The “I don’t know” response was considered as missing data. The maximum score of the scale is 32 points. Higher scores denote higher impact.

An additional section included a general parental perception of their children’s oral health and its relationship to the general wellbeing was added at the end of the MIS-EC [20].

### Translation and cross-cultural adaptation

The translation and cross-cultural adaptation and validation of the Arabic version of the MIS-EC (MIS-EC/Ar) version adopted WHO’s relevant guidelines [25]. Cross-cultural adaptation was preceded by concept review to examine the conceptual and theoretical framework of the original questionnaire for cultural relevance. Rigorous cross-cultural adaptation is considered essential for ensuring the validity and comparability to the original MIS-EC and ensure that the MIS-EC/Ar accurately measures the intended construct, hence enhancing the generalizability of MIS-EC.

Cross-cultural adaptation was performed on the original English version of the MIS-EC, and it went into five phases. The first phase was item translation. Arabic translation of the English version of MIS-EC was performed by two independent translators with health-care professional background to synthesize two drafts of MIS-EC/Ar. The translators are native Arabic speakers with excellent English command. One of them is a dentist to ensure comprehensive understanding of the questionnaire related aspects. Both translators are culturally competent, being able to understand and address the values, beliefs, health behaviors, and potential cultural biases of Arabic nations. Cultural appropriateness was ensured by employing commonly used Arabic terminology and idiomatic expressions rather than literal translations, thereby maintaining conceptual equivalence while ensuring comprehensibility for the target population. To ensure translation fidelity, an additional Arabic translation was independently generated from the Brazilian Portuguese version using Google Translate, providing a third draft of MIS-EC/Ar for comparison and validation purposes.

In the second phase, the translators examined the three Arabic drafts, addressed any discrepancies to reach consensus, then one translation was synthesized and labelled translated-MIS-EC/Ar.

The third phase comprised back-translation of translated-MIS-EC/Ar. Back-translation was done by two fluent bilingual (English/Arabic) independent translators with medical background. The two back-translations showed no conceptual conflict with the translated-MIS-EC/Ar.

The fourth phase was a final review and conciliation of all the translations to reach consensus on discrepancies. The conciliation produced the pre-final version MIS-EC/Ar.

The fifth phase of cultural adaptation was pre-testing the pre-final version by a small sample of the target population to assess comprehension, clarity, and cultural appropriateness. In this phase, thirty caregivers not included in the main study were tested by a one-to-one interview with the examiner. They were asked to identify any misunderstood words. In this phase, the “often” response translation was rephrased to be clearer to the target population. In addition, in-depth cognitive interviews with a small sample of four caregivers were conducted to explore the participants’ understanding and interpretation of the questionnaire questions. Cultural adaptation based on the findings of pre-testing and cognitive interviews produced the final version of MIS-EC/Ar.

After accomplishing cross-cultural adaptation, a question on the comprehensibility of the Arabic version of the questionnaire was added to test the comprehensibility in larger sample size.

### Participation and recruitment

This study was conducted in the outpatient clinic of the Pediatric Dentistry Department of the Faculty of Dentistry, Cairo University, Cairo, Egypt. The study followed convenient sampling where all patients presenting to the clinic fulfilling the study inclusion criteria during the study period were enrolled. The inclusion criteria were: (1) children aged between 3 and 6 years, (2) free of any systemic diseases or physical or mental disabilities, (3) with no pain related to dental caries. Exclusion criteria included: (1) the history of either dental trauma or orthodontic therapy, (2) children with any permanent dentition, and (3) non-Arabic speaking parents/caregivers. Participants were recruited from September 2024 to November 2024.

### Sample size

The appropriate sample size to assess reliability during questionnaire validation should be at least 30 or five-folds the number of questionnaire items, i.e., 40 respondents [26, 27]. The appropriate sample size to perform confirmatory factor analysis during construct validity is generally acceptable to be between 150 and 300 [28]. Thus, the sample size for this study was 200.

### Outcomes and measures

All preschoolers’ mothers/caregivers who sought treatment in the outpatient clinic of the Pediatric dentistry department of the Faculty of Dentistry, Cairo University, Cairo, Egypt, were recruited. They took the questionnaire by direct interview. The oral examination followed the decayed, missing, and filled teeth (dmft) caries index according to the WHO criteria [29]. Malocclusion was assessed based on the standards developed by Foster et al. [30], where children with anterior openbite or increased incisor overjet >3 mm or anterior crossbite or posterior crossbite were classified as children with malocclusion. Each malocclusion class was given a score ranging from zero (having no malocclusion) to 23 (having class 4l malocclusion) [31]. These scores are used for discriminant and criterion validity assessment.

### MIS-EC/Ar validation

MIS-EC/Ar validation included reliability and validity testing. Reliability was assessed based on the internal consistency using Cronbach’s alpha and test-retest reliability. Internal consistency was evaluated using Cronbach’s alpha, with values ≥ 0.7 considered acceptable for research purposes. Standardized Cronbach’s alpha coefficients were calculated to account for the differing numbers of items across domains [32]. For test-retest reliability, a random sample of thirty mothers/caregivers of participants received the MIS-EC/Ar for a second time after 2-weeks interval, and intraclass correlation coefficient (ICC) was calculated and interpreted as: <0.50 indicating poor reliability, 0.50–0.74 indicating moderate reliability, 0.75–0.89 indicating good reliability, and ≥0.90 indicating excellent reliability.

The validity testing covered content, construct, and criterion validity. Content validity was established at the stage of the original MIS-EC synthesis through expert review. Construct validity included convergent, discriminant, and structural validity. Criterion validity was assessed by examining the relationship between MIS-EC/Ar scores and the degree of malocclusion using Foster and Hamilton’s classification system, which categorizes malocclusion on an ordinal scale from 0 (no malocclusion) to 23 (Class 4l malocclusion) using Spearman’s correlation test [30, 31].

Correlations were interpreted as in the original scale, where r < 0.20 indicates poor correlation, *r* = 0.21–0.40 indicates fair correlation, *r* = 0.41–0.60 indicates moderate correlation, *r* = 0.61–0.80 indicates good correlation, and *r* ≥ 0.81 indicates excellent correlation [20].

Convergent validity was assessed through Spearman’s correlation coefficients among one of the following scores at one side: total score, child impact section score, or family impact section score, and the two general questions at the other side. Correlations were assessed as in the criterion validity, where moderate, good, and excellent correlations (*r* > 0.4) were considered a sign of related measures. While poor to fair correlations of *r* ≤ 0.4 indicated unrelated measures [33].

Comparison of the scores of participants with and without malocclusion was used to assess discriminant validity. This comparison was conducted using Mann‒Whitney U test.

Structural validity was assesssed using exploratory and confirmatory factor analysis aimed to explore and confirm the factors that significantly contribute to the measurement of the impact of malocclusion on the preschoolers’ OHR-QoL. Methodological details of exploratory factor analysis are summarized in the Supplementary material.

### Intra-examiner agreement

For clinical calibration through assessment of intra-examiner agreement, oral examination was repeated for thirteen participants after one week. Cohen’s Kappa value was 1 (*p* < 0.001), indicating absolute agreement between the two occasions.

### Statistical analysis

Statistical analysis was performed using SPSS software 27.0 (IBM Corp., NY, USA) at a statistical significance level of 0.05. Descriptive data were reported as frequencies, while quantitative data were reported as median (range).

### Ethical approval

This study was approved by the Research Ethics Committee of the Faculty of Dentistry, Cairo University, Cairo, Egypt, with reference number 46/9/24

## Results

### The study participants

In the initial phase of the study from September to October 2024, 210 patients were recruited. Due to the small proportion (16.2%) of children having malocclusion, the study launched a second phase of recruitment where only malocclusion patients (*n* = 26) were recruited in November of the same year. Participants completed the questionnaire in approximately 10 min. Participants’ demographic, socioeconomic, and general characteristics are presented in Table 1.

**Table 1:** Participants’ characteristics (*n* = 236) expressed as percentages or median (range).

Male gender	54.7%
**Age**	4 (3-6)
**Malocclusion**	25%
**Parental top education**	
Non/illiterate	7.3%
Primary	9.8%
Preparatory	25.6%
Secondary or equivalents	39.7%
Graduate	17.5%
Post-graduate study	0%
**Family income**	
<National minimum	35%
1–2.5 the national minimum	52.1%
2.5–4 the national minimum	11.1%
4–10 the national minimum	1.7%

Only 1.7% (*n* = 4) of participants stated that they faced some extent of difficulty understanding the questions of MIS-EC/Ar.

### Reliability statistics

#### Internal consistency

The total, unstandardized Cronbach’s alpha value for the MIS-EC/Ar was 0.760, indicating appropriate internal consistency. Table 2 describes Cronbach’s alpha values if any of the eight questions of the MIS-EC/Ar were deleted. The deletion of any of the eight questions would not enhance the questionnaire’s reliability. Therefore, all eight questions of the original version were kept in the Arabic version.

**Table 2:** MIS-EC/Ar reliability analysis; Cronbach’s alpha if item was deleted (*n* = 236).

	Item	missing records - “I Do not Know” responses	Mean	Variance	Corrected item-total correlation	Cronbach’s Alpha if deleted
**Child impact domain**	**1- Difficulty in eating or biting**	**0–3**	0.42	3.097	0.451	0.738
	**2- Difficulty in pronunciation**	**0–23**	0.42	3.258	0.375	0.752
	**3-Bad temper**	**1–0**	0.42	2.928	0.699	0.687
	**4-Shyness or embarrassment**	**1–0**	0.48	3.777	0.510	0.747
	**5- Smiling avoidance**	**1–0**	0.48	3.815	0.377	0.754
	**6- Being bullied**	**1–0**	0.45	3.396	0.579	0.722
**Family impact domain**	**7- Guilt or annoyance sensation**	**0–0**	0.36	2.345	0.627	0.712
	**8-Financial load**	**0–0**	0.47	3.596	0.385	0.747

Domain level internal consistency (Table 4) was acceptable for the child impact domain and the entire questionnaire, whose standardized Cronbach’s alpha was 0.797, indicating proper internal consistency.

#### Test-retest reliability

The results of test-retest reliability are shown in Table 3 at item-level and Table 4 at domain level. At item level, ICC values ranged from 0.79 to 1, indicating good to excellent reliability except item number 2 (ICC 0.481), indicating poor test-retest reliability. Test-retest ICC of the total questionnaire and its domains exceeded 0.8 at a significance level of *p* < 0.001, indicating excellent test-retest reliability of the entire MIS-EC/Ar.

**Table 3:** MIS-EC/Ar reliability analysis: test-retest reliability at item level (*n* = 30).

Item	Excluded record *N* (%)	Average ICC	ICC 95% confidence interval	*P*
**Item-1**	1 (3.2%)	1.000	NA	NA
**Item-2**	2 (6.5%)	0.481	-0.097 - 0.756	0.044
**Item-3**	1 (3.2%)	1.000	NA	NA
**Item-4**	1 (3.2%)	1.000	NA	NA
**Item-5**	0 (0%)	NA^a^	NA	NA
**Item-6**	0 (0%)	0.889	0.769–0.947	<0.0001
**Item-7**	0 (0%)	0.788	0.548–0.896	<0.0001
**Item-8**	0 (0%)	1.000	NA	NA

*ICC* intraclass correlation coefficient.

^a^Zero variance item.

**Table 4:** MIS-EC/Ar reliability analysis: internal consistency and test-retest reliability at domain-level.

Domain	Items’ count	Internal consistency	Test-retest reliability	
		Cronbach’s alpha^a^ *N* = 236	ICC (95% CI) *N* = 30	*p*
**Child impact**	6	0.756	0.962 (0.937–0.980)	<0.001
**Family impact**	2	0.445	0.839 (0.720–0.916)	<0.001
**Total**	8	0.797	0.929 (0.884–0.962)	<0.001

^a^On standardized items.

*ICC* intraclass correlation coefficient, *CI* Confidence interval.

### Criterion validity statistics

Spearman correlation analysis was conducted to test the relationship between the questionnaire total score and the degree of malocclusion the child suffers. The yielded Spearman’s correlation coefficient of 0.298 (*p* 0.01).

### Construct validity statistics

#### Convergent validity

The results of convergent validity assessment are shown in Table 5. All correlations had *r* < 0.4, indicating that overall oral health and general wellbeing were unrelated to the MIS-EC/Ar domains.

**Table 5:** Correlation between the MIS-EC/Ar domains’ score and the overall oral health or general wellbeing.

		**Total**	**Child impact**	**Family impact**
**Overall oral health** ***n*** = **236**	**Spearman correlation**	0.159^a^	0.110	0.187^b^
	***P***	0.015	0.092	0.004
**General wellbeing** ***N*** = **229**	**Spearman Correlation**	0.100	0.074	0.112
	***P***	0.130	0.267	0.090

^a^Significant difference as *P* < 0.05.

bSignificant difference as *P* < 0.01.

#### Discriminant validity

Table 6 presents the comparison of scores of the study participants with and without malocclusion, showing a statistically significant difference between the two groups at both the child impact and family impact domains in addition to the total score.

**Table 6:** Discriminant Validity results comparing the scores of children with and without malocclusion expressed as mean (range).

	Malocclusion group	No malocclusion group	*P*^a^
**Child impact**	0.66 (0–9)	0.21 (0–8)	0.003^b^
**Family impact**	0.47 (0–4)	0.11 (0–8)	0.001^b^
**Total score**	1.14 (0–13)	0.32 (0–16)	<0.001^b^

^a^Comparisons were conducted using Mann‒Whitney U test.

^b^Significant difference as P < 0.01.

#### Structural validity

Kaiser-Meyer-Olkin (KMO) measure of the base model, including all eight items, was 0.515, which falls below the generally recommended threshold of 0.60, indicating suboptimal sampling adequacy for factor analysis. However, Bartlett’s test of sphericity was statistically significant (*p* < 0.001), confirming that the correlation matrix differed significantly from an identity matrix and that factor analysis could proceed with caution. Communalities exceeded 0.50 for all items, supporting item retention for exploratory analysis.

The base model had three components collectively explained 71.1% of the variance. However, the rotated component matrix revealed problematic factor structure characterized by cross-loadings and split-loadings for multiple items (items 1, 3, 4, and 7), indicating conceptual overlap between factors [34]. This finding suggested ambiguity in how respondents interpreted certain items, potentially reflecting either multidimensional item content or sample-specific response patterns.

To achieve a more interpretable factor structure, we conducted stepwise forward deletion of cross-loading or split loading items, prioritizing models that maintained >60% variance explanation while maximizing KMO values. Detailed results of this iterative process are provided in the Supplementary Material.

The optimal reduced model retained six items, items 2,3,5,6,7 and 8, with a KMO of 0.598, explaining 65.03% of the variance across two components. Post-rotation, components 1 and 2 explained 32.6% and 32.4% of variance, respectively, as shown in Table 7. Component 1 was primarily defined by item-6 (loading = 0.773), item-8 (loading = 0.823), and item-7 (loading = 0.672), representing family impact and self-esteem/social interaction domains. Component 2 was characterized by item-2 (loading = 0.608), item-5 (loading = 0.828), and item-3 (loading = 0.789), representing functional and psychological/social child impact domains. While this model showed improved factor structure compared to the base model, residual cross-loadings remained for items 3 and 7, suggesting ongoing item overlap that should be addressed in future scale refinement.

**Table 7:** Rotated component matrix of the best model^a^.

	Component 1	Component 2
**Item-2**	0.025	**0.608**
**Item-5**	0.054	**0.828**
**Item-6**	**0.773**	0.210
**Item-8**	**0.823**	−0.108
**Item-3**	0.477	**0.789**
**Item-7**	**0.672**	0.461

Primary loading is indicated in bold.

^a^Extraction Method: Principal Component Analysis, Rotation Method: Varimax with Kaiser normalization, Rotation converged in 3 iterations.

Confirmatory factor analysis (CFA) could not be conducted since the responses of participants to item-3 and item-5 did not include all possible Likert scale categories. This restriction in the range of responses indicates that these items may not be capturing the full spectrum of malocclusion impact in the studied population, representing a limitation that warrants further investigation with larger and more diverse samples.

## Discussion

Malocclusion is a highly prevalent oral health disease in children [4]. Malocclusion can affect oral functions such as mastication and speech, as well as dental-maxillofacial growth, besides its effect on the facial appearance and psychological health of the child [2]. The rapidly growing notion of the OHR-QoL and the better understanding of the effect of oral diseases on the quality of life, as well as general health, have led to developing patient-centered measures of oral health status [11]. Multiple-item questionnaires are commonly used to assess OHR-QoL based on subject/family perception for the physical and psychological health [35]. OHR-QoL studies for children less than 6 years old usually recruit parents or primary caregivers who are capable of abstract thinking and reasoning, unlike the children at this age [17]. The effect of malocclusion on OHR-QoL in preschoolers is a relatively emerging field [15, 16, 20]. Previous studies showed controversy on the impact of malocclusion on OHR-QoL in preschool children using the Early Childhood Oral Health Impact Scale (ECOHIS), which is not a specific scale for malocclusion [36–38]. The recently designed MIS-EC addressed the effect of malocclusion on OHR-QoL. MIS-EC scale has been validated only in Brazilian Portuguese, English, Chinese, and Hindi languages [15, 16, 20, 39].

The validation of the questionnaire after proper translation into another language and cultural adjustments is important to allow comparisons with different countries using the same scale [40]. The aim of the present study is to validate an Arabic version of the MIS-EC scale to explore the effect of malocclusion on OHR-QoL in Arabic-speaking preschool children. The validation process conducted in this study followed relevant WHO guidelines [25].

Reliability analysis of MIS-EC/Ar is represented by Cronbach’s alpha value and ICC. The non standardized total Cronbach’s alpha value of 0.760, exceeding the commonly acceptable cut-off of 0.7, indicates acceptable internal consistency [32]. Internal consistency means that the questionnaire items are relatively homogeneous and are likely measuring the same construct of malocclusion. The Cronbach’s alpha value for the original MIS-EC was 0.87 [20], that of the Chinese version was 0.943 [15], while that of the Hindi version was 0.71 [39]. Having less Cronbach alpha than the original and the Chinese versions does not indicate less reliability [32]. Differences in reported Cronbach’s alpha may be attributed to different cultural or social backgrounds, as reliability estimates can vary across language adaptations and sociocultural contexts. For example, a reliability generalization meta-analysis of the Patient Health Questionnaire-9 (PHQ-9) demonstrated that Cronbach’s alpha coefficients varied substantially across different cultural and linguistic adaptations (ranging from 0.46 to 0.95), with factors such as sample heterogeneity, educational level, and cultural conceptualizations of depression contributing to this variability [41]. Similarly, differences in internal consistency estimates between the MIS-EC/Ar and other language versions likely reflect the complex interplay of cultural perceptions of malocclusion, oral health literacy, and social desirability responses in the Egyptian context.

The domain level internal consistency analysis yielded a Cronbach’s alpha of 0.756, 0.445 for the child impact, and family impact, respectively, while the standardized Cronbach’s alpha of the entire scale was 0.797, indicating good internal consistency of the MIS-EC/Ar. The low family impact domain’s Cronbach’s alpha may have been affected by the small number of questions [32] that comprise this domain, which is only two questions. Yet, it did not impair the internal consistency of the entire scale.

Test-retest reliability was tested at item, domain, and entire scale levels. The item-wise assessment of the test-retest reliability revealed that four items had absolute reliability, two had good reliability, but one had poor test-retest reliability, which is the second question that focuses on the effect of malocclusion on speaking (ICC of 0.481, 95% confidence interval (CI) −0.097 to 0.756, *P* = 0.044). However, the upper boundary of its CI exceeded the limit of good test-retest reliability of ICC > 0.75. Parental awareness may have impacted the responses to item-2 since some parents may lack the ability to differentiate between delayed or impaired speech due to malocclusion versus other causes at the studied age group of 3–6 years old. The poor test-retest reliability of item-2 did not hinder the domain level test-retest reliability that remained good with ICC of 0.962 (95% CI of 0.937–0.980, *P* < 0.001).

The ICC of the test-retest reliability of the entire MIS-EC/Ar was excellent, 0.929 (CI 0.884–0.962), and was comparable to original MIS-EC (0.94). Like the original scale, we conducted the retest after two weeks [20]. These results are comparable to other validated versions of the MIS-EC: the Chinese version [15] reported an ICC of 0.873, while that of the Hindi version was 0.87 [39]. The consistency across different cultural adaptations suggests that the temporal stability of the MIS-EC construct is maintained despite linguistic and cultural variations.

Criterion validity yielded a fair correlation (*r* = 0.298, *p* 0.01) between the total score and the degree of malocclusion, indicating that the MIS-EC/Ar is a fair measure of malocclusion and it should be combined with other diagnostic measures such as dental examination [42]. Criterion validity assessment was not reported in the original, the Chinese, or the Hindi scale. Therefore, the present study provides novel evidence regarding the relationship between MIS-EC scores and objective malocclusion severity.

Convergent validity assessed the relationship of the MIS-EC/Ar scores and general wellbeing, and oral health perception as captured by the two additional items. General wellbeing showed no significant correlation with total or domain scores (all *r* < 0.4, *p* > 0.05). The weak correlation between general wellbeing and MIS-EC domains observed in our study aligns with findings from the Hindi version [39], but contrasts with stronger correlations reported in the original and the Chinese versions [15, 20].

The total score and the family impact domain score were poorly correlated (*r* < 0.4, *P* < 0.05) to the overall oral health perception of the participants. Interestingly, while the Hindi version similarly reported weak correlations (*r* < 0.40, *p* > 0.05) between domain scores and overall oral health perception, they observed an excellent correlation (*r* = 0.94, *p* < 0.05) between the total MIS-EC score and overall oral health [39]. This discrepancy warrants further investigation.

The weaker convergent validity in our study compared to the original and the Chinese version of the MIS-EC may reflect cultural differences. Social factors, including educational level, health literacy level, as well as cultural factors influenced by different ethnic and racial groups, could influence parental views of oral health and separate it from general health [37, 38, 42, 43]. Our sample showed a notable percentage of low educational and socioeconomic levels among participating parents that might have influenced their perception of these two general questions. Moreover, both the oral health and the general wellbeing are complex constructs that may not be adequately captured by a single question [42]. The presence of two global questions, which measure children’s oral health and the extent that their oral conditions impact their overall well-being, is presented in previous commonly used oral health-related quality of life questionnaires as the Child Perceptions Questionnaire (CPQ) [44].

The discriminative ability of the MIS-EC/Ar is consistent with that demonstrated in the original, Chinese, and Hindi versions, where all versions of MIS-EC successfully differentiated between children with and without malocclusion based on MIS-EC scores [15, 20, 39].

Structural validity as assessed by exploratory factor analysis revealed a reduced six-item model with two components structure that explained 65.03% of the variance. Both components contributed nearly equally to variance explanation. The first component is mainly dependent on family impact, along with self-esteem/social interaction (items 6–8). The second component reflected child impact at the levels of functional (pronunciation), psychological, and social (smiling) levels.

This two-factor structure differs from the original MIS-EC conceptualization, which proposed separate child impact domain of six items and family impact domain of two items [20]. The Chinese version confirmed the original two-domain structure using confirmatory factor analysis [15]. The Hindi version’s factor structure has not been published [39].

Several factors may explain the structural differences. First, the marginal KMO values (0.515 for the base model; 0.598 for the reduced model) indicate suboptimal sampling adequacy for factor analysis, suggesting that a larger sample size may be necessary to stabilize factor loadings. Noting that the total sample size of the current study (*n* = 236) exceeds that of the Chinese one (*n* = 210). Second, the residual cross-loadings in the optimized model suggest conceptual overlap in the Egyptian caregivers’ interpretation of certain items, potentially reflecting cultural differences in malocclusion impacts perception and categorization. Third, the restricted response ranges for item-3 (psychological impact) and item-5 (smiling) prevented confirmatory factor analysis [36] and may indicate that these items do not adequately capture the full spectrum of relevant experiences in the study population.

The MIS-EC/Ar scale showed significant discriminant validity, fair criterion validity, and limited convergent validity.

This study has some limitations. The marginal sampling adequacy for factor analysis (KMO = 0.598) falls slightly below the recommended threshold (0.60), potentially affecting the stability and reproducibility of the factor structure. The restricted response variability for certain items precluded confirmatory factor analysis, suggesting the questionnaire may need adaptation to fully capture malocclusion impacts experienced by Arabic preschoolers.

## Conclusion

Despite the limitations, the MIS-EC/Ar represents a valuable addition to the limited repertoire of culturally appropriate instruments for assessing malocclusion impact on OHR-QoL in Arabic-speaking preschool children. The scale demonstrated good internal consistency (standardized *α* = 0.797), excellent test-retest reliability (ICC = 0.929), and significant discriminant validity (*p* < 0.05). However, convergent validity was limited, and the factor structure differed from the original scale, suggesting cultural adaptation effects.

## Supplementary information

Supplemental material

## Data Availability

The datasets used and/or analyzed during the current study are available from the corresponding author on reasonable request.
