# Supplementary material for: Cross-cultural adaptation and validation of the Arabic version of the Malocclusion Impact Scale for Early Childhood (MIS-EC/Ar)
Source: BDJ Open. 2026 Apr 13;12:35. doi: 10.1038/s41405-026-00411-6 (PMC13077054; doi:10.1038/s41405-026-00411-6)
Supplement: Supplementary file 1 — Supplemental material [file 41405_2026_411_MOESM1_ESM.docx]

Exploratory factor analysis ^1^

Exploratory factor analysis (EFA) employed principal component analysis with varimax rotation. Model fit metrics employed during EFA included Kaiser-Meyer-Olkin (KMO) measure, Bartlett's test, and variance explained by the model. Both Bartlett's test and Kaiser-Meyer-Olkin (KMO) measure were used to assess sampling adequacy. KMO measure 0.6-0.69 indicates mediocre or acceptable factorability of the correlation matrix while KMO between 0.5-0.59 indicates miserable or barely above the minimum cut-off value. Significant Bartlett's test (p<0.05) indicates sampling adequacy for EFA. Communalities >0.3 was considered adequate for item retention. Modelling aimed to explain ≥50% of overall variance. In rotation matrix, items were considered to load strongly if their loading is >0.40 and load weakly if <0.30. Cross-loading was defined as having >0.7 loading on one factor in addition to >0.4 loading on another factor. Loading difference of 0.20 between the primary and alternative factors indicates acceptable cross-loading. Split loading was defined as divided loading with no dominant loading >0.50. Problematic split-loading comprises both loadings ≥0.40 with difference <0.20.

The base model which included all the eight items yielded a KMO measure of 0.515 and significant Barlett’s test (P<0.0001), together they indicated minimally adequate sample. Communalities were all above 0.5. and the model explained 71.1% 0f variance by its three components (Table S1).

The rotated component matrix (Table S2) revealed a complex and problematic factor structure characterized by multiple cross-loadings and split-loadings. Component 1 was primarily defined by item-5 (0.969), item-3 (0.730), and item-4 (0.628), accounting for the largest proportion of variance (42.47%). Component 2 showed strong loadings from item-6 (0.794) and item-8 (0.754). Component 3 was predominantly characterized by item-2 (0.907). However, the factor structure was compromised by substantial split-loadings and a cross-loading where item-1 exhibited split-loading across Components 2 and 3 (0.577 and 0.584, respectively), item-7 displayed split-loading across all three components (0.357, 0.553, and 0.378), and item-4 cross-loaded on Components 1 and 2 (0.628 and 0.381). Additionally, item-3 showed notable secondary loadings on Components 2 and 3 (0.324 and 0.344). These findings suggested that substantial item refinement was necessary to achieve an interpretable and psychometrically sound factor structure. Therefore, stepwise forward deletion of cross- or split-loading items was conducted to reach the model with maximum KMO and minimal of cross- or split-loadings while preserving the variance explained by the model >60%. The process of forward deletion is represented in Table S 3.

**Reference:**

1. Howard MC. A Review of Exploratory Factor Analysis Decisions and Overview of Current Practices: What We Are Doing and How Can We Improve? Int J Human–Computer Interact [Internet] 2016;32(1):51–62. Available from: https://doi.org/10.1080/10447318.2015.1087664

Table S 1: Determination of items explaining the total variance by the base model:

| **item** | **Initial Eigenvalues** | | | **Extraction Sums of Squared Loadings^#^** | | |
| --- | --- | --- | --- | --- | --- | --- |
|  | **Total** | **% of Variance** | **Cumulative %** | **Total** | **% of Variance** | **Cumulative %** |
| **1** | 3.397 | 42.465 | 42.465 | 3.397 | 42.465 | 42.465 |
| **2** | 1.262 | 15.772 | 58.237 | 1.262 | 15.772 | 58.237 |
| **3** | 1.030 | 12.870 | 71.107 | 1.030 | 12.870 | 71.107 |
| **4** | 0.824 | 10.305 | 81.413 |  |  |  |
| **5** | 0.748 | 9.348 | 90.760 |  |  |  |
| **6** | 0.366 | 4.577 | 95.337 |  |  |  |
| **7** | 0.285 | 3.560 | 98.897 |  |  |  |
| **8** | 0.088 | 1.103 | 100.000 |  |  |  |

*^#^ Extraction Method: Principal Component Analysis.*

*^$^ Varimax rotation*

Table S 2: Rotated Component Matrix of the base model:^#^

|  | **Component** | | |
| --- | --- | --- | --- |
|  | **1** | **2** | **3** |
| Q1 | -0.082 | 0.577 | 0.584 |
| Q2 | 0.210 | -0.060 | **0.907** |
| Q3 | **0.730** | 0.324 | 0.344 |
| Q4 | **0.628** | 0.381 | 0.108 |
| Q5 | **0.969** | -0.049 | 0.003 |
| Q6 | 0.336 | **0.794** | 0.001 |
| Q7 | 0.357 | 0.553 | 0.378 |
| Q8 | 0.055 | **0.754** | 0.005 |

***^#^*** *Extraction Method: Principal Component Analysis.*

*Primary loadings were indicated in bold*

The top two models were number 6 and 12. Both models have two factors and proved sample adequacy with Barlett’s test P <0.001. Model-6 has the maximum KMO measure of 0.598, explained 65.03% of the variance. Although it suffers cross- and split-loading, the items still load predominantly on one factor where item-3 and item-7 load on the first factor by difference in loading of 0.312 and 0.211, respectively. Model 12 has a KMO measure of 0.592, explained 62.68% variability has no split-loading and has one cross loading of item-7 with loading difference of 0.233.

Direct comparison between model-6 and model-12 showed that model-6 has a slightly better KMO and better variance explanation. Both models share the same number of factors and sampling adequacy. Model 12 is superior in the minimum communality of 0.513 and absence of split-loading. Model 6 was considered the best model based on its superior KMO and variance explanation at a trade-off of minor split-loading and lower minimum communality of 0.370, which is still above 0.30 threshold, since retaining more items would maintain the model’s reliability (model six’s Cronbach alpha is 0.722 and that of model twelve is 0.590).

| **Table S 3: Exploratory factor analysis; Forward deletion of cross- or split-loading items;** The best model is shaded | | | | | | | | |
| --- | --- | --- | --- | --- | --- | --- | --- | --- |
| **Items removed** | **Model number** | **KMO** | **Bartlett's test p-value** | **Total variance explained** | **Minimum communality of retained items** | **Number of components** | **Cross-loading item(s) [loadings]** | **Split- loading item(s) [loadings]** |
| **none** | 0/base | 0.515 | <0.001 | 71.1 | 0.550 | 3 | - | 1 [-0.082&0.577&0.584]  4 [0.628&0.381&0.108]  7 [0.357&0.553&0.378] |
| **Item-3** | 1 | 0.565 | <0.001 | 71.967 | 0.536 | 3 | - | 1 [0.562&-0.083&0.592]  7 [0.608&0.223&0.343] |
| **Item-1** | 2 | 0.525 | <0.001 | 61.464 | 0.324 | 2 | 3 [0.739&0.455] | - |
| **Item 7** | 3 | 0.448 | <0.001 | 74.516 | 0.592 | 3 | - | 1 [-0.057&0.592&0.623]  4 [0.630&0.434&0.158] |
| **Item-4** | 4 | 0.572 | <0.001 | 75.206 | 0.558 | 3 | - | 1 [0.557&-0.129&0.615]  7 [0.598&0.432&0.312] |
| **Items 1&3** | 5 | 0.575 | <0.001 | 60.771 | 0.294 | 2 | 4 [0.391&0.727] | - |
| **Items 1&4** | 6 | 0.598 | <0.001 | 65.033 | 0.370 | 2 | 3 [0.789 & 0.477] | 7 [0.672 & 0.461] |
| **Items 1&7** | 7 | 0.437 | <0.001 | 63.973 | 0.379 | 2 | - | 4 [0.581 and 513] |
| **Items 3&4** | 8 | 0.545 | <0.001 | 74.747 | 0.588 | 3 | - | 1 [0.544 & 0.595&-0.169]  7 [0.632 & 0.335 & 0.355] |
| **Items 3&7** | 9 | 0.507 | <0.001 | 77.090 | 0.605 | 3 | 4 [0.427&0.735 &0.230] | 1 [0.579 &-0.074& 0.630] |
| **Items 4&7** | 10 | 0.520 | <0.001 | 78.718 | 0.587 | 3 | 3 [0.799 & 0.350&0.292] | 1 [-0.101&0.581 and 0.627] |
| **Items 1&3&7** | 11 | 0.583 | <0.001 | 64.92% | 0.363 | 2 | 4 [0.529 and 0.699] | - |
| **Items 1&3&4** | 12 | 0.592 | <0.001 | 62.68% | 0.513 | 2 |  | 7 [0.688 and 0.455] |
| **Items 1&4&7** | 13 | 0.543 | <0.001 | 67.12% | 0.386 | 2 | 3 [0.805&0.444] |  |
| **Items 3&4&7** | 14 | 0.393 | <0.001 | 79.69 | 0.602 | 3 | 6 [0.824 and 0.377] | 1 [0.579 and 0.614&-0.109] |
| **Items 1 &3&4&7** | 15 | 0.432 | <0.001 | 65.342 | 0.489 | 2 | - | - |

| Table S 4: Total Variance Explained by the best reduced model: | | | | | | | | | |
| --- | --- | --- | --- | --- | --- | --- | --- | --- | --- |
| **Component** | Initial Eigenvalues | | | Extraction Sums of Squared Loadings | | | Rotation Sums of Squared Loadings | | |
|  | Total | % of Variance | Cumulative % | Total | % of Variance | Cumulative % | Total | % of Variance | Cumulative % |
| **1** | 2.770 | 46.169 | 46.169 | 2.770 | 46.169 | 46.169 | 1.957 | 32.609 | 32.609 |
| **2** | 1.132 | 18.864 | 65.033 | 1.132 | 18.864 | 65.033 | 1.945 | 32.424 | 65.033 |
| **3** | .927 | 15.455 | 80.488 |  |  |  |  |  |  |
| **4** | .584 | 9.740 | 90.229 |  |  |  |  |  |  |
| **5** | .423 | 7.048 | 97.276 |  |  |  |  |  |  |
| **6** | .163 | 2.724 | 100.000 |  |  |  |  |  |  |
| Extraction Method: Principal Component Analysis. | | | | | | | | | |
